# An Integrated Physiotherapeutic Approach With Virtual Reality in Schizophrenic Patients With Ipsilateral Femoral Shaft and Intertrochanteric Fractures: A Case report

**DOI:** 10.7759/cureus.32714

**Published:** 2022-12-19

**Authors:** Avanti A Gachake, Palash R Satone, Abhishek Daf, Om C Wadhokar, Pratik Phansopkar

**Affiliations:** 1 Department of Physiotherapy, Ravi Nair Physiotherapy College, Datta Meghe Institute of Medical Sciences, Wardha, IND; 2 Department of Musculoskeletal Physiotherapy, Ravi Nair Physiotherapy College, Datta Meghe Institute of Medical Sciences, Wardha, IND

**Keywords:** quality of life, vr, schizophrenia, femoral shaft fracture, intertrochanteric fracture

## Abstract

Femoral shaft fracture occurs through the diaphyseal region of the femur. The intertrochanteric (IT) fracture is an extracapsular fracture that occurs through the proximal metaphyseal area of the femur. The ipsilateral femoral shaft and IT fractures happen in rare cases following high-energy trauma. These fractures are difficult to manage due to their complexity. They are usually managed surgically and require prompt physiotherapeutic management postoperatively. The postoperative complications involve pain, stiffness, reduced muscle strength, deep vein thrombosis, muscular weakness, and atrophy. These complications occur primarily due to immobilization. In our case, the 30-year-old male patient was a known case of schizophrenia for three years. He met with a road traffic accident (RTA) while driving a car and acquired ipsilateral femoral shaft and IT fractures. He presented with the chief complaints of pain, swelling, deformity over the right thigh, and unable to bear weight over the right lower limb. X-rays revealed a right-side ipsilateral IT fracture and a femoral shaft fracture. The patient underwent an operation that involved open reduction and internal fixation with a proximal femoral nail under spinal epidural anesthesia. We started physical therapy management on postoperative day three. On clinical evaluation, there was a decrease in the range of motion and muscle strength of the right lower limb. This case posed us with a challenge to deliver postoperative physiotherapeutic intervention without elicitation of the symptoms associated with schizophrenia. The physiotherapy protocol involved virtual reality-based (VR-based) interventions in adjunct to conventional therapeutic interventions like strengthening exercises, range of motion exercises, application of electrical modality, balance training, gait training, VR-based relaxation, and aerobic exercises. We used the Lower Extremity Functional Scale, Manual Muscle Testing, Range of Motion, Brief Psychiatric Rating Scale, and the Numerical Pain Rating Scale as outcome measures. The comparison of pre-and post-outcome measure scores demonstrates a significant improvement.

## Introduction

Intertrochanteric fracture is the common extracapsular fracture of the proximal femur, which occurs at the level of the greater and lesser trochanters. This fracture is common in old age individuals following low-energy trauma owing to senile degenerative changes in the bone, such as osteoporosis, whereas, in young individuals, it occurs as a result of high-energy trauma [1]. About half of all femoral fractures are intertrochanteric (IT) fractures with a prevalence of 0.4% and have a peak frequency for males in their 30s and even higher for elderly females, with more than 50% of patients being over 65 [2,3]. The most common deformity patterns used to identify it are shortening, adduction and external rotation of the affected lower limb owing to the attachment of muscles that insert into the greater trochanter, namely, the gluteus medius, gluteus minimus, obturator internus, piriformis, and vastus lateralis. Anterio-posterior (AP) pelvic and lateral hip X-rays confirm the diagnosis [4,5].

Femoral shaft fractures are another variety of femoral fractures. They frequently occur from either low or high-energy trauma in older patients. The incidence of these fractures is 10 to 21 per 100,000. Two percent of these are open fractures. Femoral shaft fractures commonly require a complete trauma life support evaluation and multidisciplinary care because they are frequently associated with other comorbidities [6]. Femoral shaft fractures are mainly seen in young individuals with healthy bones because they have achieved complete bone development. The major causes of these catastrophes include falls from considerable heights, running over or crushing machinery, and high-velocity traffic accidents [7].

Ipsilateral intertrochanteric and femoral shaft fractures account for one-fourth of all femoral fractures and are unusual injuries. Surgeons face difficulties in treating this combination of fractures [8,9]. Seventy-five percent of patients are men, with an average age of 35 years. In 15% to 33% of instances, the shaft fracture is open and generally comminuted, occurring through the diaphysis of the bone [10]. Patients of intertrochanteric fractures should mobilize as soon as possible. If they do not, they might develop bedsores, urinary tract infections (UTIs), joint stiffness, pneumonia, and thrombosis. One of the key issues with managing this type of fracture is the lesser degree of the patient's return to everyday activities and capacity to carry out routine duties. About 25% of patients need long-term care. Fifty percent of patients require assistance with day-to-day activities [11].

Schizophrenia is a mental illness marked by several psychiatric symptoms, such as hallucinations, delusions, incoherent speech, disorganized or catatonic behavior, and negative feelings [12]. One key sign of schizophrenia is functional impairment, mostly related to the deterioration of psychosocial functioning [13]. Individuals with schizophrenia often show violent behavior, making it difficult to deal with in both study and therapeutic practice. Violent behavior depends on several factors, such as personality changes, a history of violent behavior toward others, deranged beliefs, auditory hallucination content, substance abuse, recklessness, suicidal behaviors, antagonism, excitement, social circumstances, age, and sex [14]. The incidence rate of fractures was 5.54 and 3.48 in individuals with schizophrenia and without any mental illness respectively. This data indicates that the incidence of fractures associated with schizophrenia is higher than those without mental illness. The long-term intake of antipsychotic medications may lead to osteoporosis, hyperprolactinemia, falls, and reduced lower limb strength which are the primary causative factors of fractures in individuals with schizophrenia [15].

## Case presentation

Patient information

**Figure 1:**
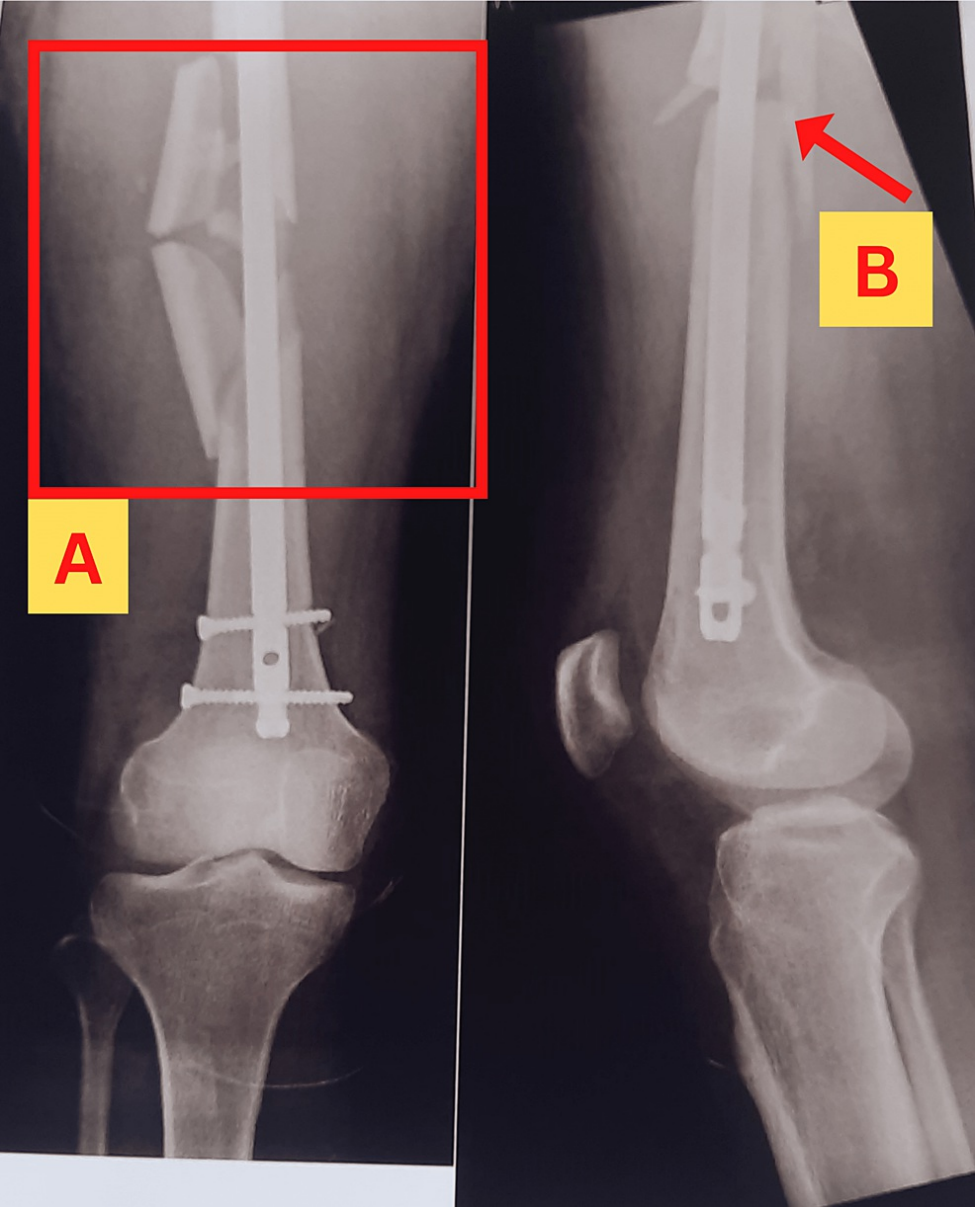
X-ray images of the shaft of the femur with PFN fixation (A) AP (anteroposterior) view; (B) lateral view Square A shows the comminuted femoral shaft fracture in the AP view, while arrow B points at the fractured femoral shaft in the lateral view. PFN: Proximal femoral nailing.

A 30-year-old male patient gave an alleged history of a road traffic accident (RTA) while driving a car. He sustained injuries to his right hip and thigh. The patient was brought to the hospital immediately by some local people who witnessed the accident. He presented with the chief complaints of pain and swelling in the groin and thigh, deformity of the right hip, and an inability to bear weight over the right lower limb. The patient gave a history that some outside force or entity was luring his car in its direction, causing it to become unbalanced and crash into a wall. An orthopedic doctor examined the patient in the emergency department and advised him for an X-ray of the affected right hip and thigh. X-rays revealed the presence of ipsilateral intertrochanteric and femoral shaft fractures on the right side, as shown in Figure 1. The patient was then shifted to the orthopedic ward where an orthopedic surgeon advised the surgery for the management of the sustained fractures. The patient underwent an operation that involved open reduction and internal fixation with a proximal femoral nail under spinal epidural anesthesia. The patient also gave a history of schizophrenia for three years for which he was on medications like amisulpride, clonazepam, and escitalopram. The initial symptoms of schizophrenia appeared in 2019. Since then, he has been wary of everyone and everything around him. He also hears voices that lead him to believe that everybody will injure or harm him. Thus, the orthopedic surgeon referred him to the psychiatry department. The psychiatrist prescribed antipsychotic medications for him. 

Clinical evaluation

**Figure 2:**
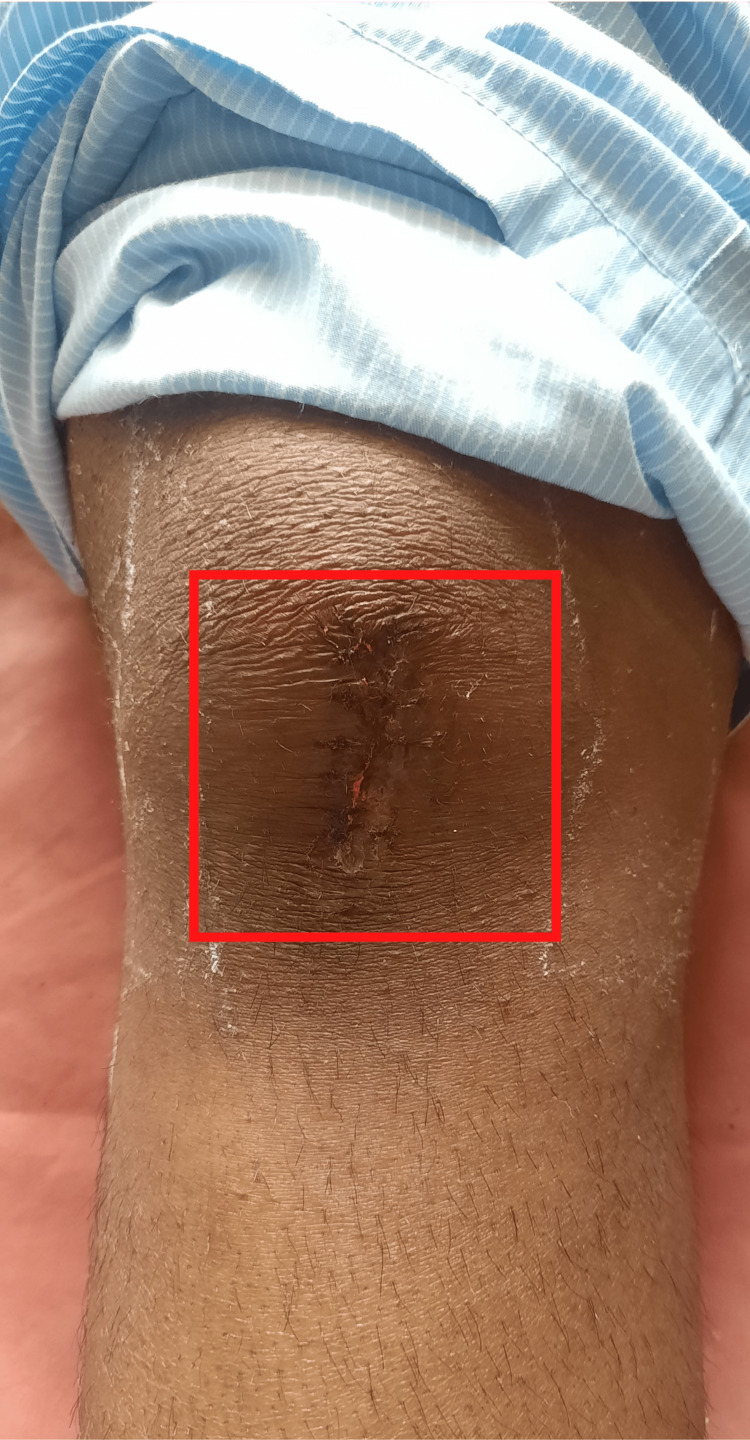
An incision scar on the aspect of the knee.

**Figure 3:**
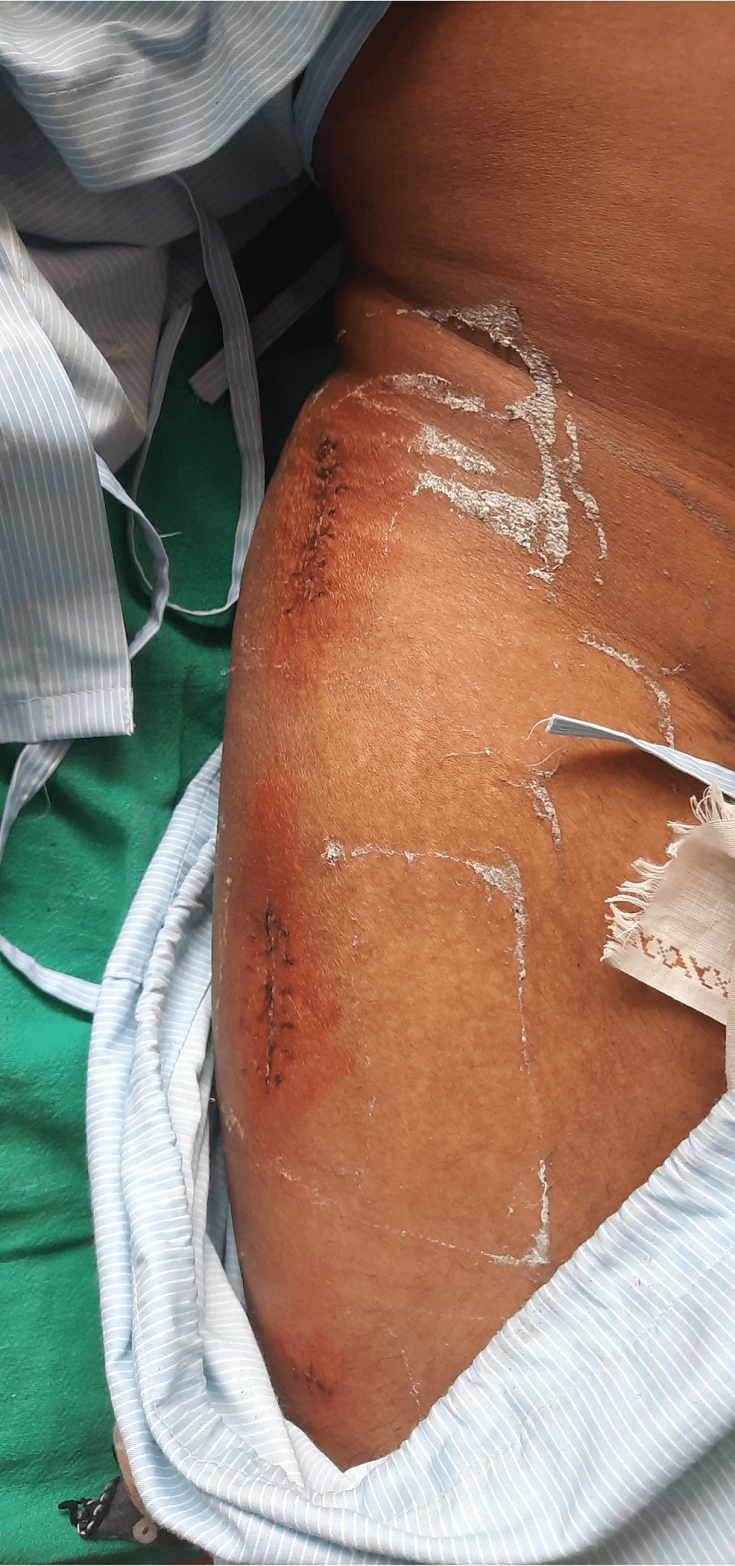
Three incision scars on the lateral aspect of the thigh.

The physical evaluation was performed on postoperative day three with the patient's and his parent's consent. The patient was evaluated in a supine lying position. Both anterior superior iliac spines (ASIS) were at the same level. On inspection, diffuse swelling was present on the right thigh. An incision scar was present on the anterior aspect of the knee, and three incision scars were on the lateral side of the thigh, as shown in (Figures 2,3). On palpation, the right thigh showed an increase in local temperature and grade 2 tenderness at the greater trochanter and over the incision scars. On examination, the dimensions of the incision scar that is present on the anterior aspect of the knee are 5 cm x 1 cm (Figure 2), and the other incision scars on the lateral side of the thigh are 1 × 1 cm, 5.5 × 2 cm, 6 × 2 cm (Figure 3).

Muscle wasting was not present (Table 1). There was a decrease in the range of motions on the affected right lower extremity (Table 2).

**Table 1:** Assessment of range of motion. ROM: Range of Motion

ROM	Right		Left	
Knee flexion	Active	Passive	Active	Passive
	20 °	30 °	110 °	120°
Knee extension	20°-0°	0-30°	110°-0 °	0-120°
Plantar flexion	35 °	40°	35°	40°
Dorsi flexion	20 °	20°	20°	20°

**Table 2:** Manual muscle testing.

Muscle	Grade
Quadriceps femoris	2-/5
Hamstrings	2-/5
Gluteus Medius	3/5

Management

**Figure 4:**
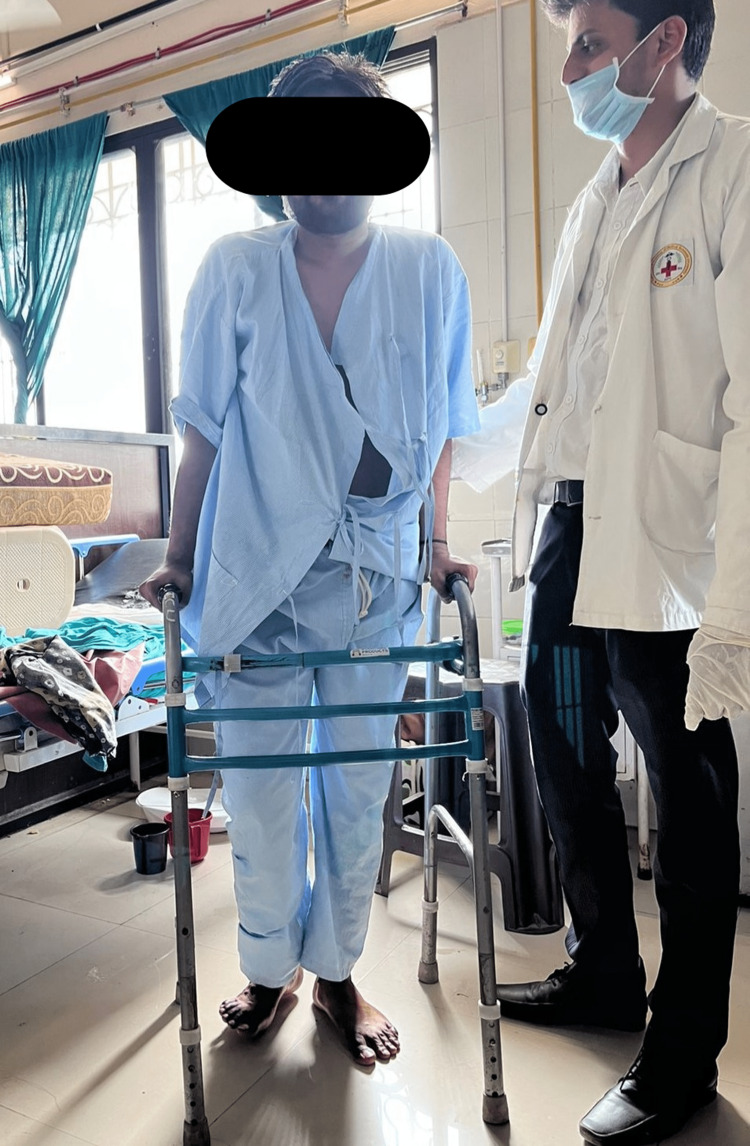
The patient is performing partial weight bearing for the affected lower limb under the observation of a physiotherapist.

The aim of the rehabilitation program is to help the patient achieve the optimal level of functional independence in activities of daily living and thereby facilitate an early return to normal life as soon as possible. The physiotherapy rehabilitation was for 12 weeks. The short-term goals of physiotherapy were to educate the patient, relax tight tissues, regain joint mobility, enhance muscular strength, avoid secondary complications, and preserve lower limb muscle strength. Secondary complications include deep vein thrombosis and pressure sores. Physiotherapy's long-term aims were to maintain the joint's functional and anatomical range of motion, improve and maintain muscle strength, and improve gait patterns. Gait training was initiated from the third week with partial weight bearing (Figure 4). It is also crucial to address the symptoms associated with schizophrenia like anxiety, agitation, and hallucination which may arise during the physical therapy session. These symptoms may interfere with the performance of given exercises and thereby restrict us from achieving the set goals in a desired duration. Thus, we advised the patient to take regular medications like amilosulpride, escitalopram, and clonazepam as prescribed by a psychiatrist. We emphasized the use of virtual reality (VR) to deliver meditation and relaxation exercises before the start of each physical therapy session.

**Figure 5:**
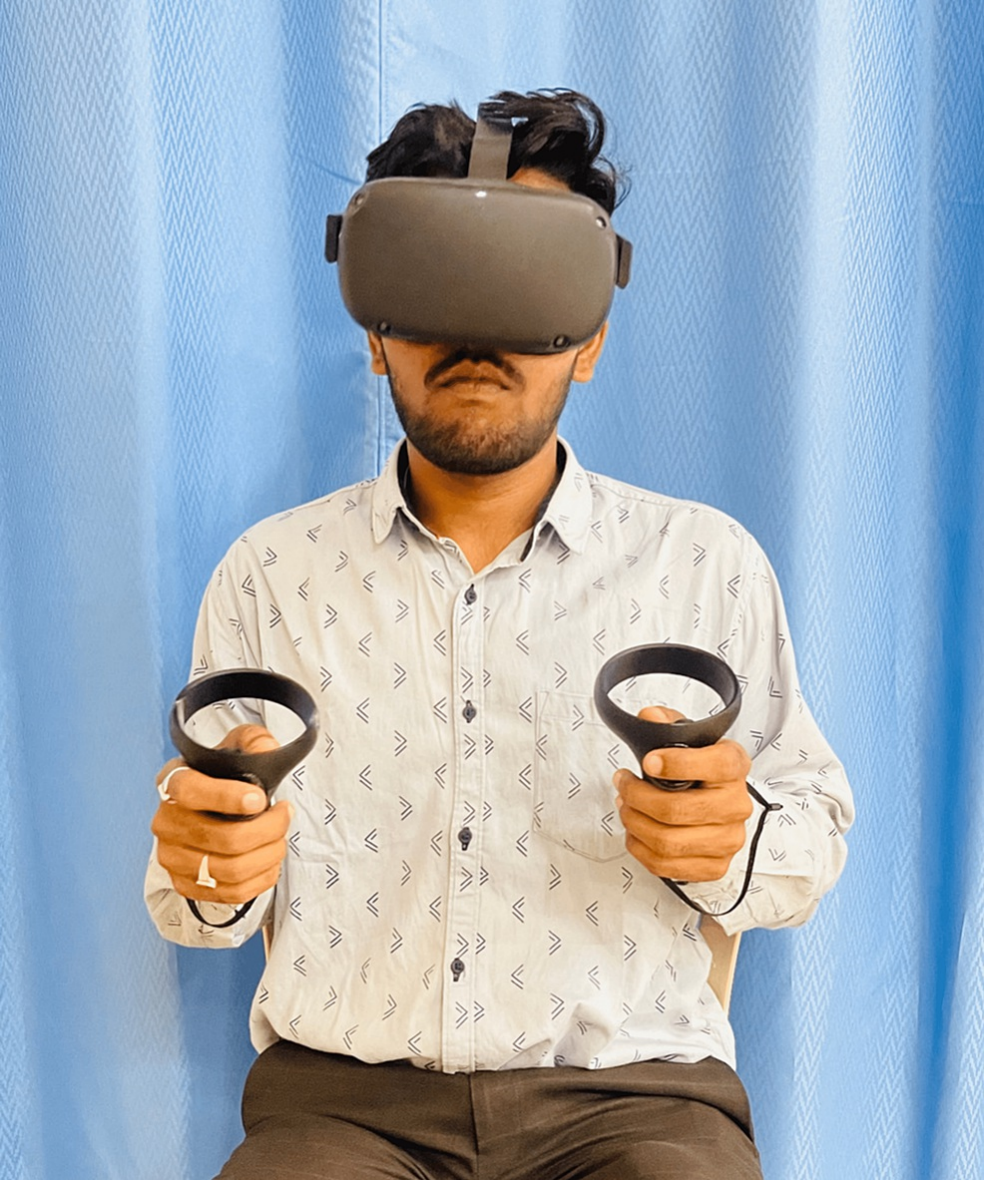
Patient doing meditation with the VR headset on. A virtual soothing and comfortable environment appropriate for meditation is created in the VR headset using an app. VR: Virtual reality

A VR app was used to create a soothing and comfortable environment in a VR headset, and then the patient was asked to meditate for 10 minutes (Figure 5). We taught the Jacobson relaxation technique to the patient with the headset still on. The environment selected for each session was of the patient's choice. Later, as the patient got habitual and comfortable with the settings and use of VR, we used certain VR games to improve the lower limb mobility, strength, balance, and gait of the patient. The summary of physiotherapy interventions provided during 12 weeks of rehabilitation is given in Tables 3-5.

**Table 3:** Summary of the applied physiotherapy interventions for weeks 1-4. ROM: Range of motion; VR: Virtual reality

Goal	Intervention	Regimen	Progression
Patient education	Educate the patient on the condition and any complications that may arise. Inform the patient about the value of physiotherapy and encourage long-term commitment to the exercise plan.	-	-
To reduce pain and swelling at the fracture site	Cryotherapy	Four to five times a day for 5-10 min.	Twice a day daily.
To improve ROM	Active ROM exercises for hip and knee joints.	10 repetitions Reciprocal inhibition 4 repetitions × 1 set	Week 2- 15 Repetition. Week 3 to 4 –20 repetitions Week 2 – 20 set 6 repetitions Week 3 and 4 – 8 repetitions
To improve the strength of the lower limb	Static strengthening exercises for quadriceps, hamstrings, and gluteal muscles. Dynamic exercises for the ankle with ½ kg weight cuff, straight leg raises, dynamic quadriceps using TheraBand or using weight cuffs	10 repetitions with a 5-sec hold	10 repetitions with a 10-sec hold 15 repetitions with a 10-sec hold 20 repetitions with a 10-sec hold
To improve the strength of the upper limb	Overhead arm flexion-extension with a thera-band. Elbow curls with 1 kg weight cuff.	10 repetitions × 1 set with yellow thera-band	15 repetitions × 1 set 20 repetitions × 1 set Green thera-band and blue thera-band
Initiation of weight-bearing on the affected leg and gait training	Pre-weight-bearing exercises such as prone lying -four-point- kneeling -knee walking. Initiate partial weight-bearing to toe-touch weight-bearing using a walker (Figure 4).	Initiate with prone lying, kneeling, and knee walking.	Initiate partial to toe-touch weight-bearing from the third week.
To reduce negative thoughts and anxiety	VR-based meditation and Jacobsen relaxation technique at the start of each session.	Meditation for 10 minutes and relaxation exercises for five minutes.	Add other casual VR games as the patient gets comfortable with the use of VR.

**Table 4:** Summarizing the physiotherapy management of weeks 5-8. ADL: Activity of daily living; VR: Virtual reality

Goal	Intervention	Regime	Progression
Improve and maintain the functional range of motion of the affected lower limb.	Assisted heel slides beyond 90. Sit with the legs hanging over the edge of the bed. Partial squats with the use of a VR game.	Assisted heel slides: Initially 10 repetitions × 1 set. High sitting: 10 minutes. Partial squats: 10 repetitions × 1 set.	At the end of the 6^th^ week. Active heel slides: 10 repetitions × 2 sets. Partial squats: 20 repetitions × 2 sets
To improve muscle strength and endurance around the hip and knee joints.	Self-resisted exercises for hip and knee muscles. VR game-assisted strengthening exercises for quadriceps and gluteal muscles.	5th week – 10 repetitions	20 repetitions × 1 set
To improve gait and gait pattern.	Full weight-bearing walking with the support of a walker. VR game-assisted gait training on the treadmill.	Initiate with five minutes of training in the fifth week.	Progress to 10 minutes of training in the sixth week
To promote mood upliftment and improve concentration.	Aerobic exercises: Initially start with seated VR games. Then progress to exercises with standing VR games. VR-assisted meditation and relaxation exercises.	10 minutes × 1 set	10 minutes × 2 sets
To improve ADL and quality of life.	Putting on and taking off clothing from the affected side, bed mobility, posture awareness with a mirror, toileting activities, and stair climbing.		

**Table 5:** Summary of physiotherapy interventions for 8 to 12 weeks. VR: Virtual reality

Goals	Intervention	Regime	Progression
To improve lower limb muscle strength	Resistance exercises with help of weight cuffs and thera-bands. VR games-assisted exercises for strengthening lower limb muscles.	Resistance exercises: 10 repetitions × 1 set. VR games-assisted exercises: 10 minutes	Resistance exercises: 10 repetitions × 2 sets. VR games-assisted exercises: 20 to 30 minutes
To improve endurance	VR games-assisted aerobic exercises: Treadmill walking and static cycling.	10 minutes	20 minutes
To improve dynamic balance and gait.	Walking on different surfaces with the VR headset on: Create a virtual environment and ask the patient to walk on different surfaces. VR games-assisted balance and gait training.	Walking on different surfaces in front of a mirror	Walking on different surfaces with VR headset on. VR games assisted-gait training
To promote mood upliftment and relaxation	VR-assisted meditation and relaxation exercises. Casual VR games.	20 minutes	Continue with the same regime

Outcome measure and follow-up

Pre-and post-interventional scores were obtained and compared following 12 weeks of rehabilitation. Outcome measures used were the Numeric Pain Rating Scale (NPRS), manual muscle testing, Range of Motion (ROM), and lower extremity functional scale. We also used Brief Psychiatric Rating Scale to keep track of the improvements in psychiatric symptoms associated with schizophrenia. Pre-and post-interventional scores are given in Table 6.

**Table 6:** Comparison of outcome measure scores after 12 weeks of rehabilitation.

Outcome measure	Pre-interventional score	Post-interventional Score
Numerical pain rating scale	9/10	2/10
Muscle strength Quadriceps femoris Hamstrings Gluteus medius	2-/5 2-/5 2-/5	4/5 4/5 4/5
Range of motion	Knee flexion: 20 degrees (active) 30 degrees (passive)	Knee flexion: 110 degrees (active) 120 degrees (passive)
Brief Psychiatric Rating Scale	104/ 168	52/168
Lower extremity functional scale	6.3%	76.3 %

## Discussion

In this case, the patient suffered an IT fracture and a femoral shaft fracture on the same limb as a result of a car collision. The primary cause of intertrochanteric fracture in young people is a traffic collision which has been treated with proximal femoral shaft nailing.

Wang et al. [9] stated that ipsilateral femoral shaft fractures and hip fractures are unusual. They account for around one-fourth of all femoral fractures and are usually associated with severe soft tissue injuries making it difficult for surgeons to deal with them. We presented a case report on a patient who sustained ipsilateral femoral shaft and intertrochanteric fractures on the right side following RTA. The comminution of the femoral shaft fracture increased the complexity that posed a challenge to deal with it.

Jawad et al. [16] and Boldin et al. [17] found in their respective studies that proximal femoral nailing (PFN) is an effective minimally invasive implant for unstable proximal femoral fractures when closure reduction is achievable. The proximal femoral nail is more biomechanically stable than the extramedullary device. Likewise, in our case, the patient was operated on with open reduction and internal fixation with PFN.

Lalwani et al. [18] concluded that early physiotherapy rehabilitation plays a vital part in the patient's recovery post-intertrochanteric fracture operated with a dynamic hip screw. It is necessary to do a range of motion exercises, strength training, and gait training. It aids in quick recovery and allows the patient to return to work. In our case, the patient had an ipsilateral femoral shaft and IT fractures. He was managed surgically by internal fixation with PFN. On postoperative evaluation, the patient demonstrated a reduced range of motion and reduced muscular strength around the hip and knee joints. We initiated isometric exercises for the weakened muscles on postoperative day three with maximum emphasis on the quadriceps, hamstrings, and gluteal muscles.

Fernández et al. [13] observed that initially, there was hesitation to use VR on patients with schizophrenia even though several researchers concluded that VR is easy to handle, entertaining, inspiring, fascinating, and did not cause anxiety in the participants. All the studies yielded encouraging outcomes in improving social skills or cognition in the short term. Some studies also found that general psychopathology, negative symptoms, and everyday activity improved after the application of VR therapy. In our case report, we encouraged the use of VR in the early phase to promote relaxation by creating a soothing environment during meditation and relaxation exercises. The patient showed enthusiasm for using the VR and gave positive feedback. The use of VR helped him boost his self-confidence and uplift his mood. The symptoms associated with schizophrenia were in control during rehabilitation due to the relaxation induced by VR-based meditation and relaxation exercises. In later stages, VR-based games were used to execute certain therapeutic exercises to improve the physical activity of the patient. 

Bisso et al. [19] stated that VR therapies are better than traditional ones because, in some circumstances, they produced results that were on par with or significantly better. It showed improvements in the follow-up after applying VR therapy for auditory-verbal hallucinations (AVH) because the behavioral patterns taught throughout the treatment were learned and reinforced in real life. Today's widespread and expanding availability of VR technology (such as the Oculus Rift or HTC Vive series) and its market adoption make it potentially an effective, affordable, and flexible therapeutic tool for schizophrenia spectrum diseases. In our case patient presented with a diagnosis of schizophrenia for three years. Thus, it presented us with a challenge to execute physiotherapeutic interventions without triggering any schizophrenia-associated symptoms. With the confidence of positive findings of previous studies on the application of VR-based rehabilitation in schizophrenia and physiotherapy setups, we implemented avatar-based VR in delivering relaxation and aerobic exercises.

Naqvi et al. [20] stated that because of modernization brought on by technical advancements, VR training programs are more accepted, exercisable, comfortable, and engaging in physiotherapy. The gamification can ease discomfort and anxiety throughout the recovery process. Since VR training programs seek to increase motion control and more actively include patients in motion learning, gamification may improve patients' functional independence of the hand and health-related quality of life (HRQoL) following distal radius fractures (DRF). Supporting this hypothesis, we delivered VR-based gaming exercise therapy in postoperative rehabilitation.

## Conclusions

Comparing the pre-and post-rehabilitation outcome measure scores after eight weeks shows significant improvements in pain, muscle strength, ROM, and lower limb function. VR-based meditation and relaxation were demonstrated to promote relaxation and reduce anxiety, depression, and hallucinations during the rehabilitation program, as reflected by the pre-and post-rehabilitation scores of the brief psychiatric rating scale. In late-stage rehabilitation, VR-based games were used to deliver certain exercises for lower limbs. This approach helped in the effective performance of exercises without the interference of schizophrenic symptoms during exercises which is reflected in the improvement of outcome measure scores. Thus, conventional physiotherapeutic interventions in adjunct to VR-based rehabilitation are proven effective in delivering relaxation and other therapeutic exercises in a patient with schizophrenia who sustained a femoral shaft fracture operated by open reduction and internal fixation with a proximal femoral nail.
